# Impact of ionizers on prevention of airborne infection in classroom

**DOI:** 10.1007/s12273-022-0959-z

**Published:** 2022-12-02

**Authors:** Chen Ren, Fariborz Haghighat, Zhuangbo Feng, Prashant Kumar, Shi-Jie Cao

**Affiliations:** 1 School of Architecture, Southeast University, 2 Sipailou, Nanjing, 210096 China; 2 Energy and Environment Group, Department of Building, Civil and Environmental Engineering, Concordia University, Montreal, H3G 1M8 Canada; 3 Global Centre for Clean Air Research (GCARE), School of Sustainability, Civil & Environmental Engineering, Faculty of Engineering & Physical Sciences, University of Surrey, Guildford, Surrey, GU2 7XH UK; 4 Institute for Sustainability, University of Surrey, Guildford, Surrey, GU2 7XH UK

**Keywords:** classroom, infection risk, ionizer, negative ions, removal efficiency

## Abstract

Infectious diseases (e.g., coronavirus disease 2019) dramatically impact human life, economy and social development. Exploring the low-cost and energy-saving approaches is essential in removing infectious virus particles from indoors, such as in classrooms. The application of air purification devices, such as negative ion generators (ionizers), gains popularity because of the favorable removal capacity for particles and the low operation cost. However, small and portable ionizers have potential disadvantages in the removal efficiency owing to the limited horizontal diffusion of negative ions. This study aims to investigate the layout strategy (number and location) of ionizers based on the energy-efficient natural ventilation in the classroom to improve removal efficiency (negative ions to particles) and decrease infection risk. Three infected students were considered in the classroom. The simulations of negative ion and particle concentrations were performed and validated by the experiment. Results showed that as the number of ionizers was 4 and 5, the removal performance was largely improved by combining ionizer with natural ventilation. Compared with the scenario without an ionizer, the scenario with 5 ionizers largely increased the average removal efficiency from around 20% to 85% and decreased the average infection risk by 23%. The setup with 5 ionizers placed upstream of the classroom was determined as the optimal layout strategy, particularly when the location and number of the infected students were unknown. This work can provide a guideline for applying ionizers to public buildings when natural ventilation is used.

**Electronic Supplementary Material (ESM):**

the Appendix is available in the online version of this article at 10.1007/s12273-022-0959-z.

### Keywords classroom;
infection risk;
ionizer;
negative ions;
removal efficiency

### 1 Introduction

The continuous invasion of infectious diseases into the indoor environment is of global concern (Nunayon et al. 2019). These infectious diseases can seriously threaten public health (Noorimotlagh et al. 2021). Currently, coronavirus disease 2019 (COVID-19) caused by severe acute respiratory syndrome coronavirus 2 (SARS-CoV-2) continues to spread at an alarming rate (Zhao et al. 2020), resulting in incalculable damage worldwide (Kumar et al. 2020). According to the knowledge about respiratory infectious diseases, two possible modes of transmission exist: direct contact (e.g., contact with the contaminated surfaces) and respiratory droplet (Kumar and Morawska 2019; Santos et al. 2020). Small respiratory droplets spread into the air and can remain airborne as aerosols (carrying the virus) for an extended period (Ai et al. 2020; Ren et al. 2021; Kumar et al. 2021). Thus, the airborne spread of infectious diseases cannot be neglected to reduce the likelihood of infection among healthy occupants (Dai and Zhao 2020; Wang et al. 2021; Berry et al. 2022).

Awareness of the factors influencing the airborne transmission mode is critical in preventing airborne infection by droplets (particularly for aerosol particles) (Niazi et al. 2021). Indoor environmental factors, including airflow rate (Kohanski et al. 2020; Ye et al. 2021) and air distribution (Ren et al. 2022b), directly affect the concentration and exposure level of airborne particles. World Health Organization (WHO 2020) and Centers for Disease Control and Prevention (CDC 2021) confirmed that the effective use of natural ventilation, such as in classrooms, can reduce the particle (carrying the viruses) concentration with low energy consumption. Previous studies (Hawendi and Gao 2018; Park et al. 2021; Qi and Wei 2021; Kumar et al. 2022) reported that the performance of natural ventilation greatly depends on the weather parameters, such as outdoor wind speed. The uncertainty of local weather conditions may lead to a lower ventilation rate than the suggested one (e.g., 30 $m^3$/h per person), further inducing an increased transmission probability of infectious diseases (Li and Tang 2021).

Therefore, American Society of Heating Refrigerating and Air conditioning Engineers (ASHRAE 2020) recommended an increased air supply of mechanical ventilation to decrease the infection risk. However, the mechanical ventilation aiming to provide a large air supply volume is challenged by high energy consumption (Ding et al. 2020; Xu et al. 2020). Sun and Zhai (2020) suggested that the ventilation rate should be increased about 15 times (from 30 to 438.2 $m^3$/h per person) of the recommended value in offices, to acquire a low infection probability. However, reaching the suggested ventilation rate is challenging for most existing ventilation systems. Thus, air purification devices (Feng et al. 2016) have been developed from the perspective of a safe environment and energy efficiency to improve the removal effect of airborne pollutants.

Air purification devices, such as air filters and ultraviolet (UV) lamps, are commonly utilized and combined with indoor ventilation systems to remove indoor pollutants (Wang et al. 2009; Abdolghader et al. 2018; Feng et al. 2021a). Air filters are based on the physical capture mechanisms of diffusion, interception, and inertia to remove indoor pollutants (Kim et al. 2021). However, the accumulation of pollutants (such as particles) may result in a high pressure drop, increased energy consumption, and replacement cost for fiber filters (Li et al. 2019). With the re-atomization and bio-release potentials, particles carrying viruses can multiply in fiber filters, further leading to secondary contamination and health risks (Nakpan et al. 2019). UV lamps are usually installed in the upper space of a room and ventilation systems. Although UV lamps can disinfect the microorganisms, they almost have no purification ability that removes the particles, such as aerosols (Fischer et al. 2020). Moreover, air filters and UV lamps are mainly applied to microenvironment; it is particularly a challenge for large space rooms to design the location of these devices, so as to effectively improve the removal performance (Zhai et al. 2021).

Negative ion generators (ionizers) have been developed to enhance the removal efficiency of particles, particularly in the large spaces (Pushpawela et al. 2017). Electrical corona discharges by negative high voltage of ionizers can decompose the neutral molecules (such as oxygen molecules) in the air, which not only produces an excess of negative ions (generally $O_2^-$ molecules), but also produces the adjunctions like positive ions (e.g., positively charged carbon dioxide) and ozone, etc. (Pushpawela et al. 2017). Negative ions electrically soon charge the particles, causing them to either repel each other or remove them by a deposition process; charged particles have a greater mobility than neutral particles and are deposited and removed more effectively (Pushpawela et al. 2017). However, a major disadvantage of ionizers is the potentially harmful effect on occupants' health (Suwardi et al. 2021); inhalation of ozone causes extensive pulmonary changes including epithelial injury and fibrosis, and positive ions have an adverse effect on human immune and nervous systems (Shargawi et al. 1999). Another concern with the introduction of ozone into indoors is the possible health impact of secondary emissions from the reaction of ozone with chemicals such as terpenes, which can produce secondary pollutants such as formaldehyde and ultrafine particles (Weschler 2000). Thus, using carbon fiber brushes in ionizers (generating the corona discharge at a relatively low applied voltage with less oxidized material and ozone emission) (Park et al. 2009) or using the ionizers in the unoccupied scenario is preferred.

Many studies illustrated that ionizers effectively remove aerosol particles from indoor chamber with different removal levels (Wu et al. 2006; Huang et al. 2008; Lai et al. 2016). The removal performance of particles almost depends on the spatial distribution of ions (Grinshpun et al. 2005). However, small and portable negative ion generators may not provide a sufficiently large removal efficiency (Shaughnessy et al. 1994; Grabarczyk 2001), because of the limited horizontal diffusion of negative ions (Pushpawela et al. 2017). The conjunction of natural ventilation and ionizer in large spaces can be effective in propagating negative ions by a longer distance, improving the removal efficiency of particles, mitigating the transmission of infectious diseases and while maintaining high energy efficiency (Srivastava et al. 2021), as shown in Figure 1. The optimal layout strategy of ionizers

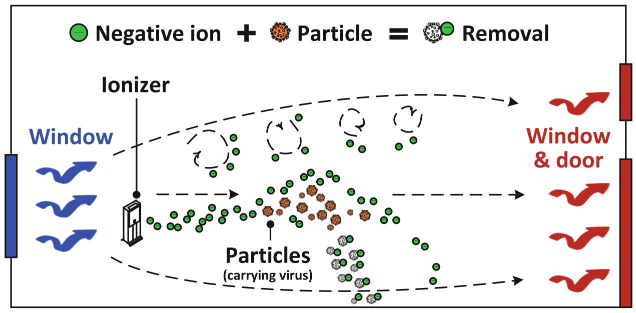

**Fig. 1** Sketch map of ionizer application based on natural ventilation should be explored to increase the removal efficiency and further reduce the infection risk, particularly during the regular prevention of the epidemic.

The present work aims to investigate the distributions of negative ions and particles in a naturally-ventilated classroom with various scenarios of ionizer layout (i.e., number and location) and infected students. The optimal layout strategy of ionizers was determined by comprehensively evaluating the removal efficiency and infection risk. This study can provide practical guidance in combining ionizers and natural ventilation, which is mainly applied to prevent airborne infection in large public buildings.

### 2 Materials and methods

The simulation method was adopted to model the distributions of negative ions and particles under different scenarios of ionizer layouts and infected students to analyze the removal performance of negative ion generators (ionizers) and the infection risk in a naturally ventilated classroom. The flowchart of this study is shown in Figure 2. The experiment validating the simulation model and estimating the removal coefficient based on a trial-and-error approach was conducted in an indoor chamber. Several simulation cases were performed to model the diffusion of negative ions and the removal of particles in the naturally ventilated classroom. The removal efficiency and infection risk were further analyzed by different evaluation models.

#### 2.1 Configuration of the model

A classroom model was constructed to investigate the distributions of negative ions and particles, the removal efficiency and the infection risk under different ionizer layouts. Based on the outdoor computational domain, the detailed geometry of a naturally ventilated classroom is illustrated in Figure 3. The classroom is on the second floor of a teaching building in Nanjing Normal University, with

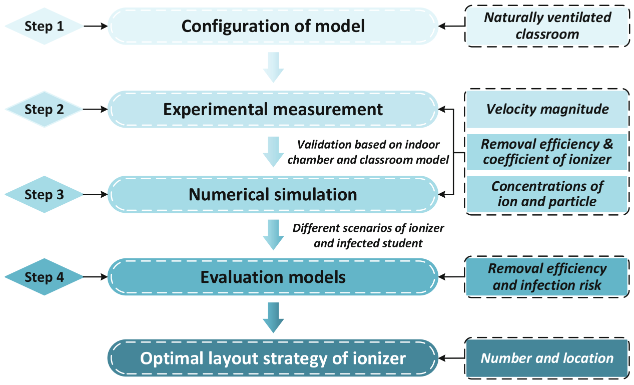

**Fig. 2** Flowchart of this work a height $H$ of 25 m (Ren et al. 2022a). The inlet is at a $5H$ distance upstream, and the outlet is at a $15H$ distance downstream. The laterals and top are at $5H$ distances away from the building. The size of the classroom is 14.0 m ($X$) × 8.5 m ($Y$) × 5.0 m ($Z$), where the left windows are located upstream of the classroom and the right windows and doors are in the downstream area. The sizes of left windows, right windows, and doors are 2.4 m ($X$) × 0.55 m ($Z$), 0.8 m ($X$) × 0.7 m ($Z$), and 1.1 m ($X$) × 2.1 m ($Z$). The $X$-axis distance between desks is 1.0 m.

#### 2.2 Experimental measurement

##### 2.2.1 *Experimental measurement in indoor chamber*

The experiments on the ion and particle concentrations and the removal efficiency of the ionizer were conducted in an indoor chamber with an area of approximately 23 $m^2$. The removal efficiency can be calculated based on the particle concentrations when the ionizers are used and not used (see Section 2.4). Figures 4 and A1 (see Appendix A, which is available in the Electronic Supplementary Material (ESM) of the online version of this paper) show the schematic of the experimental setup. An air conditioner using recirculation mode was installed on a side wall 2 m above the floor. The average supply air velocity of air conditioner was measured 2 m/s downward at 30° by a Testo-405 thermal anemometer (±0.1 m/s), which was also utilized to measure the supply air velocity of the ion nozzle and particle release device and the velocity at different monitoring locations (as presented below). In the experiment, fine particulate matter ($PM_{2.5}$) was considered dynamically similar to aerosol particle carrying virus with a diameter of less than 5 μm, which were assumed to travel in the air at a long distance before settling on surfaces (Xu et al. 2020). The measurement of the removal efficiency for the ionizer using fine particles may be overestimated because of the deposition process of particles themselves. The limitation of using fine particles is discussed in Section 4. The particle release device was used to generate the particles from candle burning. The release device was set up at the center of indoor chamber, which is an enclosed box comprising a variable frequency fan, five red columns of candles, and a release channel. Particles were emitted into indoor chamber by a release channel. The emission rate of fine particulate matters is measured as 0.5 mg/h (See and Balasubramanian 2011) for five candles. The supply air velocity of the particle release device is controlled as 1 m/s by the fan. The challenge of using this particle release device with limited quantities of candles is also discussed in Section 4.

A carbon fiber negative ion generator (ionizer) (see Appendix A in the ESM) was designed in this work with a

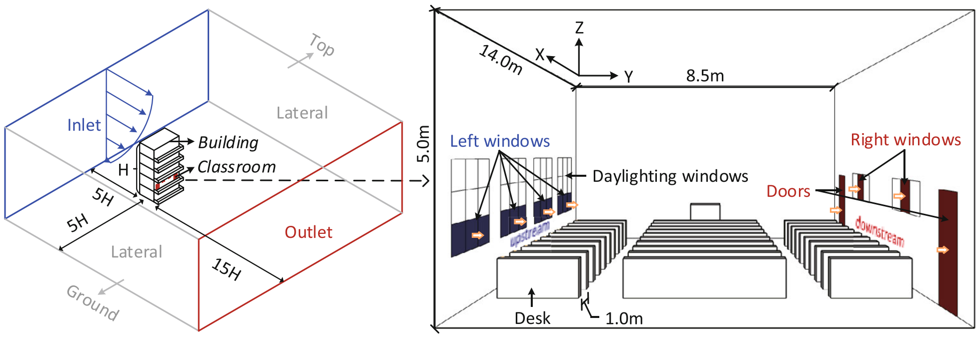

**Fig. 3** Configuration of the classroom based on outdoor computational domain

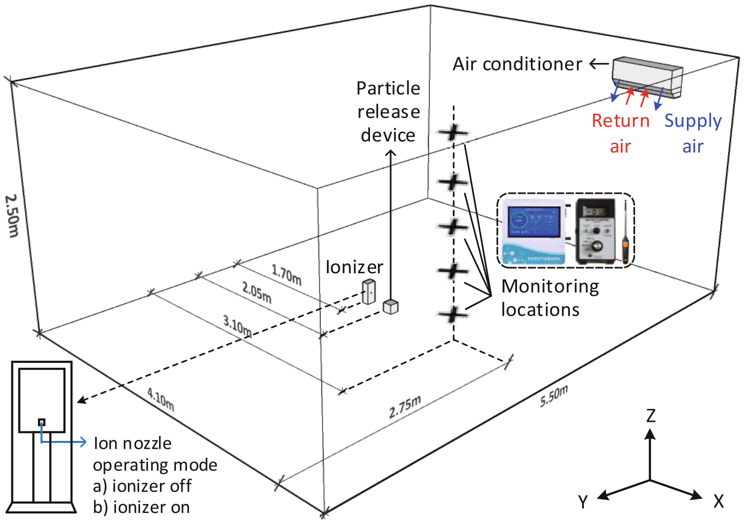

**Fig. 4** Schematic picture of experimental setup in the indoor chamber dimension of 98 mm (length) × 72 mm (width) × 225 mm (height). The maximum negative voltage of this ionizer is at −5 kV, with the measured detectable ozone concentration lower than 4 ppb; this level should still be considered negligible by the standards of most commonly used electrostatic chargers (Han et al. 2008). The ionizer was placed on the floor at (*X*, *Y*) = (1.70, 2.75) m. The ion nozzle is at the center of the ionizer to emit the ions. Table 1 lists the parameters of an ionizer, including supply air velocity of nozzle, ion generation rate, etc. The average supply air velocity of the ion nozzle is 0.1 m/s. The ion generation rate of an ionizer was measured using an air ion counter (type of Alphalab Inc AICZX21) with a unit of 1 million ions per $cm^3$ (see Appendix A in the ESM). The measurement distance was 0.1 m between the air ion counter and ionizer. The measurement period was 30 min; the average value of negative ion generation rate was obtained as 50 million #/$cm^3$, and positive ion generation was neglected. It is also a limitation of this work to neglect the specific types of ions in the experiment, which is discussed in Section 4.

The monitoring locations were located at (*X*, *Y*) = (3.10, 2.75) m with different heights of 0.4, 0.8, 1.2, 1.6, 2.0, and 2.4 m to measure the particle and negative ion concentrations and the velocity in the chamber. An air quality meter (BOYUN BYC810-FX, ± 3%) and an air ion counter were used to record the concentrations of fine particulate matters and negative ions, respectively. The particle concentrations (with the modes of ionizer on and off), negative ion concentrations (with the mode of ionizer on) and airflow velocities were recorded within a 1 h interval. The background concentrations of particles and negative ions were measured as 34 μg/$m^3$ and 3.5 million #/$cm^3$, respectively, in the indoor chamber. The experimental results of velocity, particle, negative ion, and removal efficiency in

**Table 1** Design parameters of an ionizer

| Items | Parameters |
| --- | --- |
| Method of negative ion generation | Carbon fiber brush (voltage at −5 kV) |
| Supply air velocity of ion nozzle | 0.1 m/s |
| Negative ion generation rate | 50 million #/$cm^3$ (average value) |
| Detectable ozone concentration | ≤ 4 ppb |
| Recommended coverage area | ≤ 30 $m^2$ |

the indoor chamber validated the theoretical applicability of the simulation model, as shown in Section 3.1. Estimating the removal efficiency of an ionizer using the limited monitoring locations in the indoor chamber rather than in the classroom model (shown in Figure 3) is a potential limitation, which is further discussed in Section 4.

*2.2.2 Experimental measurement in classroom*

The experimental data from Ref. (Liu et al. 2015) were used to validate the simulation results of the classroom model. The experimental monitoring of indoor velocity (DAVIS portable weather station with the resolution of 0.1 m/s and accuracy of ±5%) was performed. The monitoring locations (M1, M2, and M3) were evenly located on the desks in the classroom model (shown in Figure 5), with the coordinates of $(X, Y, Z)$ = (2.2, 4.25, 1.0) m, (6.2, 4.25, 1.0) m, and (10.2, 4.25, 1.0) m. The interval of the experimental data was 1 min. The validation of indoor velocity between experiment and simulation was performed in the previous work (Ren et al. 2022a), with an average difference of 7% (results are not shown here).

### 2.3 Numerical simulation

A computational fluid dynamics (CFD) method was used in this work to simulate the distributions of particles and negative ions. The removal performance of the ionizer and the infection risk were further evaluated. In the incompressible and steady-state simulation model, an ion is assumed to carry a negative charge (positive ion was neglected in this work) (Zhou et al. 2016). The velocity field was governed by the Re-Normalization Group (RNG) $k$-$\varepsilon$ model (Satheesan et al. 2020). The potential and electrical fields were governed by Poisson and Gaussian equations, respectively. The negative ion and particle concentrations were simulated by User-Defined Scalar (UDS) based on continuous phase model, given the assumption that particles and negative ions with a size less than 5 μm can behave like a gas compound traveling through the air at long distances (Xu et al. 2020). The scalar transport equation of ions based on potential and electrical fields is shown below.

$$\left(\boldsymbol{u}+\mu_{\mathrm{p}}\cdot E\right)\nabla n = D_{\mathrm{p}}\nabla^2 n \tag{1}$$

where, $\boldsymbol{u}$ is the air velocity (m/s); $\mu_{\mathrm{p}}$ is the ion mobility (m$^2$/(V·s)); $E$ is the electric field (V/m); $n$ is the number of negative ions (#/cm$^3$); and $D_{\mathrm{p}}$ represents the ion diffusion coefficient (m$^2$/s). The electrical force affects the charged particles by adding a momentum source term, as shown in Eq. (2). The form of governing equation for particle concentration can be displayed as shown in Eq. (3).

$$\nabla p = \nabla\cdot\left(\mu\nabla\boldsymbol{u}\right) - en\nabla\phi \tag{2}$$

$$\nabla\cdot\left[\left(\boldsymbol{u}+v_{\mathrm{s}}\right)C\right] = \nabla\cdot\left[\left(D+\varepsilon_{\mathrm{p}}\right)\nabla C\right] - AnC \tag{3}$$

where, $p$ is the pressure (Pa); $\mu$ is the dynamic viscosity (N·s/m$^2$); $e$ is the elementary charge, $1.6\times10^{-19}$ C; $v_{\mathrm{s}}$ is the settling velocity of particles (m/s); $C$ is the particle concentration (#/m$^3$); $D$ is the Brownian diffusion coefficient (m$^2$/s); $\varepsilon_{\mathrm{p}}$ is the particle eddy diffusivity (m$^2$/s); and $A$ is a constant removal coefficient (susceptibility) related to the ionizer removal efficiency (cm$^3$/#), depending on the particle size and indoor environmental factor such as temperature. Equation (3) was retrieved from modeling the removal of airborne particles by using the UV germicidal sources in an indoor environment (Zhou et al. 2016). The Brownian diffusion coefficient is defined as $2.76\times10^{-11}$ m$^2$/s (Hinds 2012), and the settling velocity of fine particles is determined as $3\times10^{-5}$ m/s (Wei and Li 2015).

The value of $A$ can be estimated by a trial-and-error approach, which was validated by Zhou et al. (2016). First, the initial value of removal coefficient $A$ was defined to simulate the removal efficiency of the ionizer. A new value of removal coefficient was tried when the deviation of removal efficiency between experimental and simulation results was above 5%. In this study, the estimation of the removal coefficient for the ionizer was conducted in the indoor chamber. Using the fine particles in the removal coefficient estimation rather than aerosol particles is hypothesized due to their similarity to small particles, such as those that are less than 5 μm (Xu et al. 2020).

The overview of simulation cases and boundary conditions regarding the estimation of removal coefficient and the validation of removal efficiency of an ionizer in the indoor chamber is shown in Table 2. The turbulent intensity and turbulent viscosity ratio for the velocity-inlet were set as 5% and 10, respectively. Cases 1 and 2 were used to estimate the removal coefficient and validate the removal efficiency with the experimental results. Moreover, the grid independence among coarse (573,421), medium (894,105) and fine (1,532,214) grids was analyzed under the scenarios of Cases 2–4. The convergence of governing equations was assumed when the residuals were less than $10^{-6}$. The diffusion and convection terms were discretized by the second-order schemes. The SIMPLE algorithm is considered to couple pressure and velocity fields.

Different ionizer layouts (number and location) and three infected students were considered to simulate the negative ions, particles, removal efficiency of the ionizer, and infection risk in the classroom model. Figure 5 shows the locations of infected students (S1, S2, and S3) and ionizers (G1–G8). The ionizers were placed close to the left windows (upstream), and the ion nozzle faced the right windows. The middle rows of desks are preferred by the students. Thus, the ionizers were mainly placed in the middle area of the room, and only one ionizer was considered in the first or last row. The layouts of ionizers in the middle area or downstream of the classroom were also considered, as shown in Appendix B (in the ESM). With a total height of 1.28 m and a mouth size of 0.02 m × 0.02 m, the detailed model for the scenario with infected student is displayed in Figure 5. The infected student was considered to be coughing at an average rate of 13 m/s downward at 27.5° (Li et al. 2021). The body temperature of students (with clothing) was set at 20 °C (Ren et al. 2022a). The coughing temperature was assumed to be 36 °C (Ren et al. 2021). The intensity of particles exhaled from an infected student was set as 1 × $10^{-4}$ #/$m^3$ (Leng et al. 2020), which was used as a reference particle concentration ($C_{ref}$). The overview of simulation cases under different scenarios (A, B, C, D, E, and F) of infected students and ionizers upstream of the classroom are listed in Table 3.

**Table 2** Overview of the simulation cases in the indoor chamber

| Case No. | Boundary conditions | Notes |
|---|---|---|
| 1 | Air conditioner: velocity-inlet with 2.0 m/s downward at 30°, outflow<br>Ion nozzle: velocity-inlet with 0.1 m/s<br>Particle release device: velocity-inlet with 1.0 m/s | Number of grids: 894,105 |
| 2 | Air conditioner: velocity-inlet with 2.0 m/s downward at 30°, outflow<br>Particle release device: velocity-inlet with 1.0 m/s | Number of grids: 894,105 |
| 3 | Same as Case 2 | Number of grids: 573,421 |
| 4 | Same as Case 2 | Number of grids: 1,532,214 |

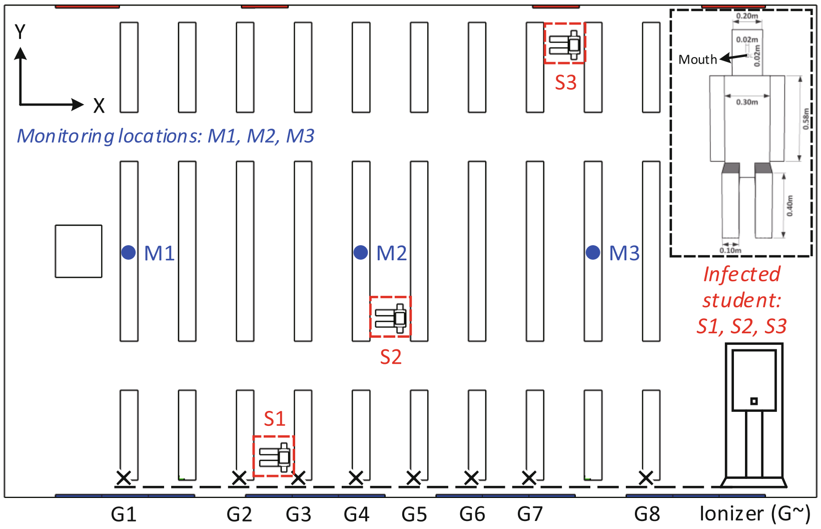

**Fig. 5** Layout of infected students, ionizers at the upstream area (indicated by × symbol), and monitoring locations (indicated by ● symbol) in the naturally ventilated classroom

**Table 3** Overview of the simulation cases under different scenarios of infected students and ionizers (upstream) in the naturally ventilated classroom

| Scenario | Case No. | Location of the ionizer upstream of the classroom | Number of ionizers | Location of infected students |
|---|---|---|---|---|
| A | 5–7 | None | 0 | S1, S2, S3 |
| B | 8–10 | G4 | 1 | Same as scenario A |
| C | 11–13 | G3 + G6 | 2 | Same as scenario A |
| D | 14–16 | G1 + G3 + G6 | 3 | Same as scenario A |
| E | 17–19 | G1 + G3 + G6 + G8 | 4 | Same as scenario A |
| F | 20–22 | G1 + G2 + G4 + G6 + G8 | 5 | Same as scenario A |

Note: The layouts of ionizers in the middle area and downstream of the classroom are shown in Appendix B in the ESM.

In the outdoor computational domain with the classroom model (see Figure 3), the grid number of 7,288,548 for unstructured mesh was verified to predict the velocity; the unstructured mesh differs from the fine grids with a grid number of 16,419,830 by less than 5% (Ren et al. 2022a). The inlet was defined as velocity-inlet, and the inflow profile of wind speed followed the power-law type wind model with a power-law exponent of 0.25 (Ren et al. 2022a). The reference wind speed was set as 0.23 m/s at the height of 10 m (Ren et al. 2022a). The outlet is set as outflow, and the top and lateral boundaries were set as symmetry. The geometric roughness height for the ground was set as 1.3 m considering that the roughness height constant is 7 (Weerasuriya et al. 2019). The turbulent intensity and turbulent viscosity ratio for the inlet were 5% and 10, respectively. The building walls, classroom desks, and body surfaces of students were defined as the non-slip wall. The supply air and wall temperature was set at 20 °C. In the simulation cases using the classroom model, the governing equations were assumed to converge as the residuals are below $10^{-6}$. The second-order schemes and the SIMPLE algorithm were also utilized.

### 2.4 Evaluation models

In this work, the removal efficiency of ionizers in the room (i.e., indoor chamber and classroom) is calculated based on the particle concentrations in the air when the ionizers or natural ventilation are used (or ionizers and natural ventilation are combined) and when the ionizers and natural ventilation are not used. The removal efficiency can be defined as follows.

$$\eta = \left(1 - \frac{C_{\text{on}}}{C_{\text{off}}}\right) \times 100\% \tag{4}$$

where, $\eta$ is the removal efficiency (%); $C_{\text{on}}$ is the particle concentration (#/m$^3$) in the air when the ionizers or natural ventilation are used (or ionizers and natural ventilation are combined); and $C_{\text{off}}$ is the particle concentration (#/m$^3$) in the air when the ionizers and natural ventilation are not used.

On the basis of spatial distribution of particles, the infection risk was further analyzed under different scenarios in terms of the infected students' location. According to the Wells-Riley equation, the average quantum concentration can be considered in the steady-state simulation if the quantum generation rate, breathing rate, and the number of infectors and people in the room remain constant during the exposure time (Rudnick and Milton 2003). The evaluation model of infection risk as a function of time-averaging particle concentration ($\bar{C}$, #/m$^3$) and exposure time ($T$, h) was rewritten according to Buonanno et al. (2020), as shown below.

$$R = \left(1 - \exp\left(-\text{IR} \times \bar{C} \times T\right)\right) \times 100\% \tag{5}$$

where, $R$ is the infection risk (%); IR is the inhalation rate of the exposed student (m$^3$/h), which can be defined as 0.96 (m$^3$/h) (Buonanno et al. 2020); and $T$ was set as 1 h in this work.

## 3 Results

In this section, the removal coefficient of the negative ion generator (ionizer) was estimated. The velocity, particle and negative ion concentrations and removal efficiency were validated between the experiment and simulation results. The spatial distributions of negative ions and particles were simulated in the classroom model to assess the removal performance under different scenarios of ionizer layouts and infected students. The removal efficiency and infection risk were evaluated for the optimal layout strategy of ionizers.

### 3.1 Validation of experiment and simulation

The removal efficiency of an ionizer in the indoor chamber was measured to estimate the removal coefficient [as shown in Eq. (3)] and validate the simulation model. The removal coefficient was estimated at $1 \times 10^{-7}$ cm$^3$/# by a trial-and-error approach. The measured values of velocity, particle concentration, and negative ion concentration (when the ionizer is on) at the monitoring location were also used to validate the simulation.

Figure 6 compares the CFD and experimental results of normalized velocity magnitude ($U/U_{\max}$) and particle concentration ($C/C_{\max}$) when an ionizer is on and off at the monitoring location in the indoor chamber. The simulation

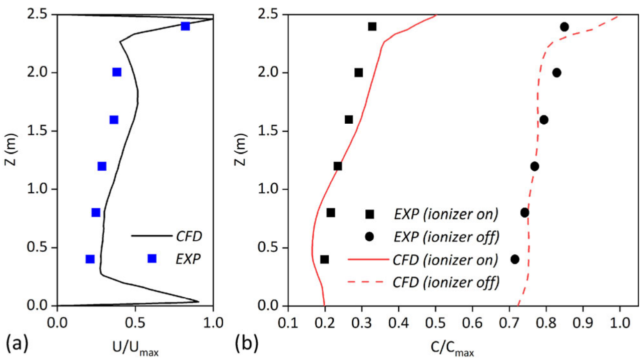

**Fig. 6** Comparisons of normalized velocity magnitude ($U/U_{\max}$) and particle concentration ($C/C_{\max}$) between experiment and CFD simulation at the monitoring location in the indoor chamber: (a) velocity, (b) fine particle concentration with the mode of ionizer on and off and experimental results agreed well in terms of velocity and particle concentration. The calculated maximum deviations were 8.5% and 14.3% for velocity and particle concentration, respectively. Compared with the mode of ionizer off, the mode of ionizer on achieved a particle removal rate of 52%.

Figure 7 compares the simulation and experiment results of the negative ion concentration (with the mode of ionizer on) and removal efficiency in the indoor chamber. At the monitoring location, the negative ion concentration predicted by the simulation model agreed well with the experimental result with an average difference of 2%. The average removal efficiencies obtained from experiment and simulation for an ionizer in the indoor chamber both approached 70%.

Compared with the experiment, the simulation model based on the estimated removal coefficient can perform well in predicting the removal performance of the ionizer. In Cases 2–4, the differences of velocity and particle concentration between the medium grids and fine grids were less than 5%, proving the feasibility of medium grids in the simulation.

### 3.2 Influence of ionizer layout on negative ion and particle distributions

The natural ventilation performance based on the window opening mode in Figure 3 was verified in the previous work (Ren et al. 2022a) with the Air Diffusion Performance Index (ADPI) approaching 70%, proving the well-mixed airflow distribution for natural ventilation in the classroom. Then, the influence of ionizer layout on the distribution of negative ions was further analyzed.

Figure 8 shows the distribution of negative ions at the plane of $Z$ = 1.1 m under different scenarios of ionizers (see Table 3). The negative ions were diffused in the room along the upstream supply air. The negative ion concentration

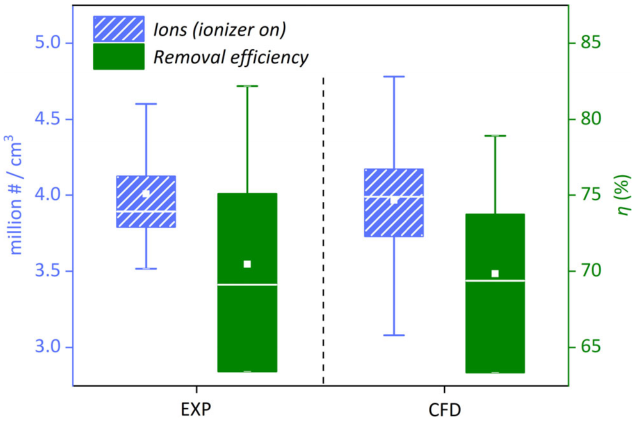

**Fig. 7** Comparisons of negative ion concentration (million #/cm$^3$) and removal efficiency (%) of an ionizer between experiment and CFD simulation at the monitoring location in the indoor chamber

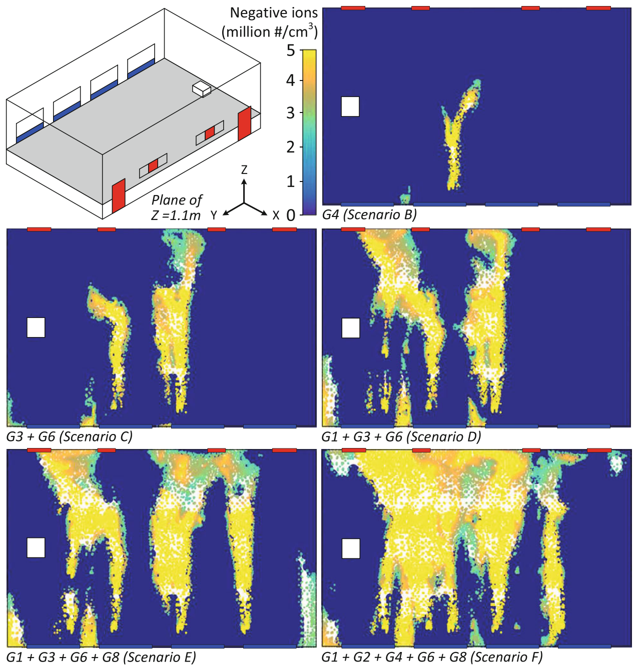

**Fig. 8** Distribution of negative ions (million #/cm$^3$) at the plane of $Z$ = 1.1 m with different scenarios of ionizers (upstream) in the naturally ventilated classroom decreased as the distance from the ionizers increased. The maximum negative ion concentration approached 5 million #/cm$^3$. As the number of ionizers increased, the spatial accessibility of negative ions was enhanced in the air. When the number of ionizers was 4 and 5, the coverage percentage of negative ions reached more than 60% in the desk area at the breathing plane.

The influence of ionizer layout (number and location) on the removal of particles was analyzed under different scenarios of infected students. Figures 9–11 display the distributions of particles at the plane of $Z$ = 1.1 m under different scenarios of ionizers (upstream) and infected students at locations S1, S2, and S3 (scenarios A–F in Table 3). The particle distribution was dependent on the negative ion concentration. The removal effect of particles was enhanced as the number of ionizers increased. The particles were almost distributed in the front classroom when the infected student was at S1. The coverage percentage of particles reached 40% in the desk area when no ionizer was installed. As the ionizer number increased to 1, 2, and 3, the coverage percentage decreased to 33%, 28%, and 20%, respectively, in the desk area. Compared with the scenario without an ionizer, the scenarios with 4 and 5 ionizers reduced the average relative particle concentrations ($C/C_{\text{ref}}$), by 19% and 44%, respectively.

As seen in Figure 10, the particles were nearly distributed in the middle of the classroom when the infected student was at S2. Compared with that when the infected student was at S1, the coverage percentage of particles when the infected student was at S2 increased by 8%–20% in the desk area. The reason can be attributed to the increased distance between the infected students and ionizers, and numerous ions were inactivated because of their short life span. The average relative particle concentration was calculated as 2.2% ($C/C_{ref}$) when the particles were not exposed to ions. Compared with the average particle concentration when no ionizer was installed, the average relative particle concentrations when the number of ionizers was 4 and 5 were decreased by 41% and 51% ($C/C_{ref}$), respectively.

Compared with the particle concentration when the infected student was at S1 and S2, the particle concentration at the breathing plane when the infected student was at S3 increased, as shown in Figure 11. Without an ionizer in the classroom, the average relative particle concentration was 3.5% ($C/C_{ref}$) at the breathing plane of 1.1 m. The removal effect of negative ions to particles was improved with the infected student at S3 when the ionizer number increased to 4 and 5. Thus, with an unknown infected student in the classroom, the ionizer number of 5 (at the upstream of the classroom) can show excellent performance in reducing particle concentration based on natural ventilation. As shown in Appendix B (in the ESM), the layout of ionizers located at upstream of the classroom was more favorable than other layouts (in the middle area or downstream of the classroom).

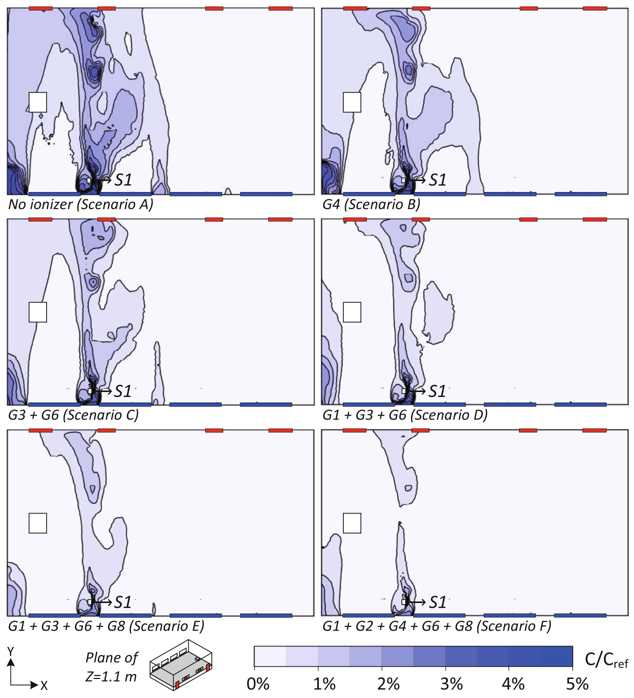

**Fig. 9** Contour of relative particle concentration ($C/C_{ref}$) at the plane of $Z$ = 1.1 m under infected student of S1 with different scenarios of ionizers (upstream) in the naturally ventilated classroom

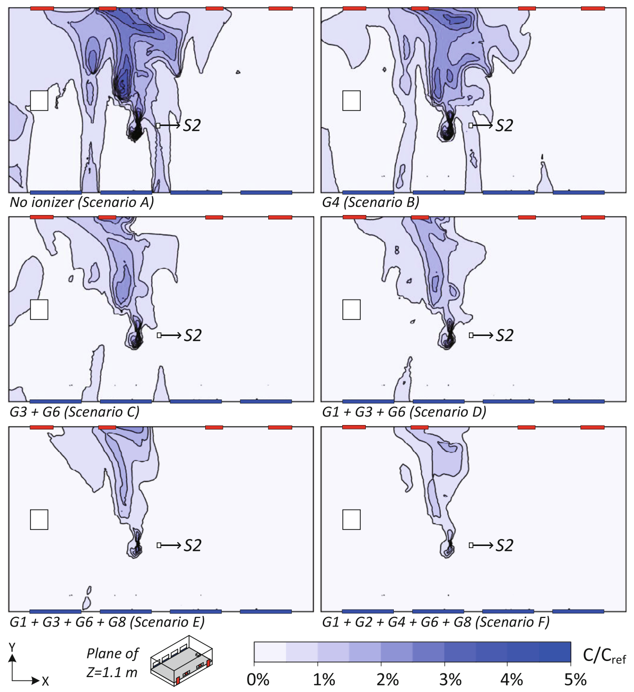

**Fig. 10** Contour of relative particle concentration ($C/C_{ref}$) at the plane of $Z$ = 1.1 m under infected student of S2 with different scenarios of ionizers (upstream) in the naturally ventilated classroom

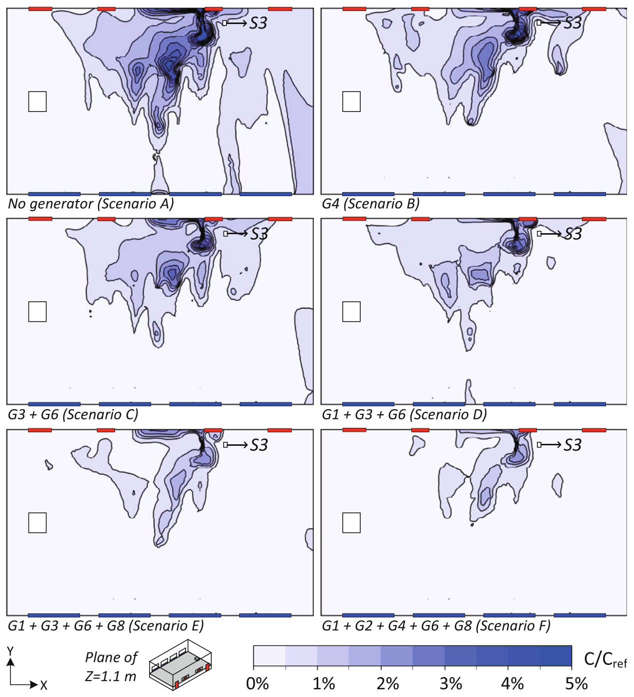

**Fig. 11** Contour of relative particle concentration ($C/C_{ref}$) at the plane of $Z$ = 1.1 m under infected student of S3 with different scenarios of ionizers (upstream) in the naturally ventilated classroom

#### 3.3 Influence of ionizer layout on removal efficiency and infection risk

The influence of ionizer layout (location and number) on removal efficiency and infection risk in the classroom was analyzed based on the distributions of particles, to determine the optimal layout strategy. The removal efficiency of independently using the ionizer (upstream of the classroom) is also shown in Appendix C (in the ESM). As regards the infected students of S1 + S2, S1 + S3, S2 + S3, and S1 + S2 + S3, linear ventilation model (LVM) was used to obtain the particle distributions rapidly (Cao and Meyers 2014), and evaluate the removal efficiency and infection risk.

Figure 12 illustrates the removal efficiency under various scenarios of ionizer number (0–5) and infected students (S1, S2, S3, and S1 + S2 + S3), based on natural ventilation. The removal efficiency with two infected students (S1 + S2, S1 + S3, and S2 + S3) and different numbers of ionizers (0–5) is demonstrated in Appendix D (in the ESM). The removal efficiency was obtained based on the particle concentrations in the breathing region of $Z \leq 1.1$ m. Compared with independently using the ionizers (see Appendix C in the ESM), the combined use of ionizers and natural ventilation increased the removal efficiency. The removal efficiency approximately increased linearly as the ionizer number increased. When there was no ionizer (using natural ventilation independently), the average removal efficiencies were below 20%. When the ionizer number was 1, the average removal efficiency was around 20% with infected student at S1, S2, and S3; removal efficiency was below 20% with two infected students. The minimum removal efficiency was 10% when the number of infected students was three. The average removal efficiency for the scenarios with infected student at S1, S2, S3, and S1 + S2 + S3 was enhanced by 17%, 35%, 43%, and 63% as ionizer number increased from 1 to 2, 3, 4, and 5, respectively. Five ionizers could contribute to the comprehensive removal of particles in the air, particularly when the location of the infected student changed.

Figure 13 illustrates the infection risk (exposure time of 1 h) when the infected student was at S1, S2, S3, and S1 + S2 + S3 with different numbers of ionizers (0–5). The infection risk was calculated by the particle concentration at the breathing plane of $Z = 1.1$ m. The average infection risk was reduced largely by 23% when the number of ionizers increased from 0 to 5. The infection risk when the infected student was at S1 was lower than that when the infected student was at S2 and S3, because of the closer distance from ionizers. The average infection risk under the scenario of a single infected student was minimally reduced to 13.4% (when the ionizer number was 5 and the infected student was at S1). The average infection risk with three infected students was minimally reduced to 21.2% when the ionizer number was 5.

Tables 4 and 5 show the significant difference analysis based on $p$-value of removal efficiency and infection risk, respectively, under different numbers of ionizers (0–5). With the increased number of ionizers, the effectiveness of ionizers was improved (in terms of removal efficiency and infection risk) since the $p$ value was gradually decreased less than 0.01. The feasibility of using ionizers with the number of 5 was well proved when compared to the scenario without using the ionizer. It also seems to be no significant difference between using 3 and 4 ionizers, mainly because of G8 location of ionizer (ref., Figure 5) away from infected

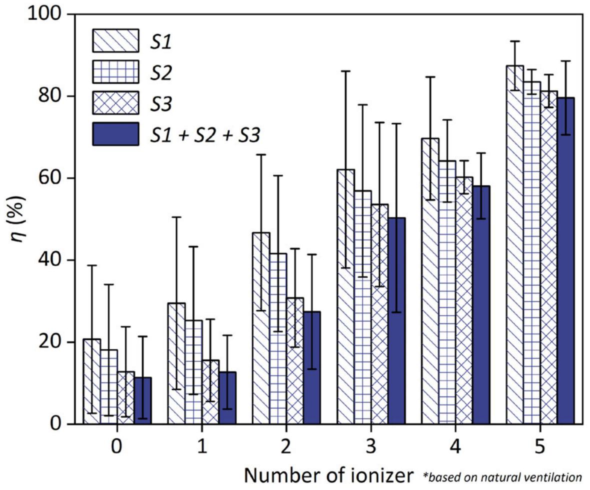

**Fig. 12** Removal efficiency under different numbers of ionizers (0–5) at the upstream area and different scenarios of infected students (S1, S2, S3, and S1 + S2 + S3) in the naturally-ventilated classroom

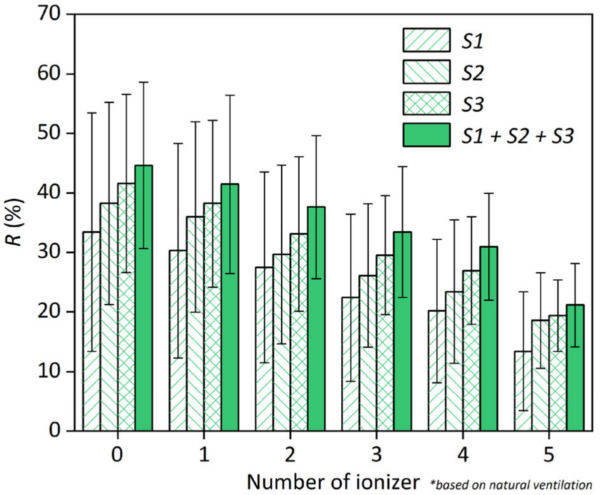

**Fig. 13** Infection risk (with the exposure time of 1 h) under different numbers of ionizers (0–5) at the upstream area and different scenarios of infected students (S1, S2, S3, and S1 + S2 + S3) in the naturally-ventilated classroom students with potentially weaker removal performance compared to other locations of ionizer. Tables 6 and 7 show the significant difference analysis based on $p$-value of removal efficiency and infection risk, respectively, under different locations of infected students (S1, S2, S3, and S1 + S2 + S3) and different numbers of ionizers (1–5). We found that the differences of removal efficiency and infection risk were significant for the scenarios of different locations of infected students ($p < 0.01$). Hence, in terms of various locations of infected students, the effectiveness of ionizers

**Table 4** Significant difference analysis based on $p$-values of removal efficiency under different numbers of ionizers

| Number of ionizers | 0 | 1 | 2 | 3 | 4 | 5 |
|---|---|---|---|---|---|---|
| 0 | 1 | 0.38 | 1.00E−01 | 5.09E−06 | 7.87E−10 | 6.26E−14 |
| 1 | | 1 | 0.02 | 5.94E−05 | 3.83E−08 | 4.71E−12 |
| 2 | | | 1 | 0.02 | 7.40E−05 | 4.13E−09 |
| 3 | | | | 1 | 0.25 | 1.21E−04 |
| 4 | | | | | 1 | 4.45E−03 |
| 5 | | | | | | 1 |

**Table 5** Significant difference analysis based on $p$-values of infection risk under different numbers of ionizers

| Number of ionizers | 0 | 1 | 2 | 3 | 4 | 5 |
|---|---|---|---|---|---|---|
| 0 | 1 | 0.62 | 0.20 | 0.04 | 2.32E−03 | 1.98E−04 |
| 1 | | 1 | 0.42 | 0.11 | 0.04 | 6.35E−04 |
| 2 | | | 1 | 0.41 | 0.17 | 3.54E−03 |
| 3 | | | | 1 | 0.57 | 4.25E−03 |
| 4 | | | | | 1 | 5.47E−03 |
| 5 | | | | | | 1 |

**Table 6** Significant difference analysis based on $p$-values of removal efficiency under various locations of infected students (S1, S2, S3, and S1 + S2 + S3) and different numbers of ionizers

| Number of ionizers | Location of infected student | S1 | S2 | S3 | S1+S2+S3 |
|---|---|---|---|---|---|
| 1 | S1 | 1 | 8.05E−03 | 3.58E−03 | 2.72E−03 |
| | S2 | | 1 | 4.59E−03 | 3.39E−03 |
| | S3 | | | 1 | 7.29E−03 |
| | S1+S2+S3 | | | | 1 |
| 2 | S1 | 1 | 7.58E−03 | 2.88E−03 | 2.29E−03 |
| | S2 | | 1 | 4.53E−03 | 3.56E−03 |
| | S3 | | | 1 | 7.63E−03 |
| | S1+S2+S3 | | | | 1 |
| 3 | S1 | 1 | 7.92E−03 | 6.61E−03 | 5.72E−03 |
| | S2 | | 1 | 8.52E−03 | 7.32E−03 |
| | S3 | | | 1 | 8.63E−03 |
| | S1+S2+S3 | | | | 1 |
| 4 | S1 | 1 | 6.25E−03 | 3.54E−03 | 3.03E−03 |
| | S2 | | 1 | 5.57E−03 | 4.56E−03 |
| | S3 | | | 1 | 7.01E−03 |
| | S1+S2+S3 | | | | 1 |
| 5 | S1 | 1 | 3.71E−03 | 2.14E−03 | 2.79E−03 |
| | S2 | | 1 | 4.79E−03 | 5.15E−03 |
| | S3 | | | 1 | 7.85E−03 |
| | S1+S2+S3 | | | | 1 |

**Table 7** Significant difference analysis based on $p$-values of infection risk under various locations of infected students (S1, S2, S3, and S1 + S2 + S3) and different numbers of ionizers

| Number of ionizers | Location of infected student | S1 | S2 | S3 | S1+S2+S3 |
|---|---|---|---|---|---|
| 1 | S1 | 1 | 7.06E−03 | 5.81E−03 | 4.55E−03 |
| | S2 | | 1 | 8.64E−03 | 6.85E−03 |
| | S3 | | | 1 | 7.96E−03 |
| | S1+S2+S3 | | | | 1 |
| 2 | S1 | 1 | 8.72E−03 | 6.62E−03 | 4.31E−03 |
| | S2 | | 1 | 7.80E−03 | 5.14E−03 |
| | S3 | | | 1 | 6.82E−03 |
| | S1+S2+S3 | | | | 1 |
| 3 | S1 | 1 | 7.43E−03 | 5.11E−03 | 3.44E−03 |
| | S2 | | 1 | 7.24E−03 | 4.82E−03 |
| | S3 | | | 1 | 6.75E−03 |
| | S1+S2+S3 | | | | 1 |
| 4 | S1 | 1 | 7.57E−03 | 4.79E−03 | 2.81E−03 |
| | S2 | | 1 | 7.06E−03 | 4.34E−03 |
| | S3 | | | 1 | 6.13E−03 |
| | S1+S2+S3 | | | | 1 |
| 5 | S1 | 1 | 5.22E−03 | 4.25E−03 | 3.34E−03 |
| | S2 | | 1 | 8.98E−03 | 6.99E−03 |
| | S3 | | | 1 | 7.57E−03 |
| | S1+S2+S3 | | | | 1 |

was validated on reducing indoor pollutant concentration and mitigating the transmission probability.

From the perspective of removal efficiency and infection risk, 5 and the upstream, i.e., G1, G3, G4, G6 and G8 (see Figure 5), were suggested as the number and locations for the optimal layout strategy of ionizers when the infected student was unknown in this naturally ventilated classroom with an area of 119 $m^2$. With the precondition of an average removal efficiency of more than 80% and average infection risk of less than 20% (only with one infected student), the optimal coverage area by an ionizer was estimated as 23.8 $m^2$, slightly lower than the recommended value of 30 $m^2$.

### 4 Discussion

The optimal layout of negative ion generators (ionizers) was investigated in the naturally ventilated classroom, to increase the removal efficiency and reduce the infection risk. The ionizers combined with the natural ventilation were more effective at different levels (i.e., from 20% to 80% of average removal efficiency; average infection risk reduced from 40% to 13%) than natural ventilation or ionizers used independently (see Appendix C in the ESM). However, ionizers can also harm human health, with the potential positive ion and ozone generation. Using the ionizers in the unoccupied scenarios, such as nighttime or outdoor activities, is preferred. Moreover, the intention of this study is to investigate the potential use of ionizers in classrooms, with the main focus of numbers and layout. It is suggested to use the ionizers without the appearance of students considering their side effects on health.

In this study, the key factor of the ionizer number on the removal efficiency and infection risk was analyzed. The ionizer number of 5 (upstream of the classroom) showed excellent performance in reducing the particle concentration based on natural ventilation. With a negative ion emission rate of 50 million #/$cm^3$ and outdoor wind speed of 0.23 m/s, an ionizer was estimated to contribute to approximately 0.15% of average removal efficiency per meter square (%/$m^2$), and reducing 4.6% of average infection risk per meter square (%/$m^2$). The power of an ionizer was measured as 5.4 W by a power monitor (DL333501C-1). When the ionizer number was 5, the total energy consumption per unit of floor area ($m^2$) was calculated 0.23 W/$m^2$, which can be negligible when compared with the energy consumption of a conventional air conditioning system (37 W/$m^2$) (Xu and Gao 2022).

The results also showed that the removal efficiency decreased when the distance was far away from the ionizers because of the limited horizontal diffusion of negative ions. The location in which the ionizers were upstream of the classroom was favorable. This finding is validated in Appendix B (in the ESM). Moreover, the emission rate of the ionizer considerably affected the removal efficiency of particles. Small and portable ionizers could not provide a sufficiently large emission rate of negative ions according to Adachi et al. (1993). Thus, in this work, the combination of natural ventilation and portable ionizers had the advantage of enhancing the spatial accessibility of negative ions in unoccupied environment and improving the removal effectiveness of particles, particularly during the regular prevention of infectious diseases; the optimal design of ionizers involving the installation of a low-cost fan inside can also be convenient.

The limitations of this study and future work are discussed as follows. The exact mechanism for the positive ion and ozone production by corona discharges of a carbon fiber ionizer remains unclear. This issue should be further considered to minimize the harmful effect of positive ions, ozone, and secondary pollutants on human health by taking measures, such as using a DC corona discharge module based on dielectric barrier effect in the ionizers, or coating the polypropylene film on the ground electrode of discharging device to reduce the concentrations of adjunctions (such as ozone and positive ion) (Lee et al. 2020). For the experimental work, the estimation of the removal coefficient of an ionizer should be performed in the actual environment using a highly sophisticated monitoring system, considering various types of specific ions and airborne pollutants, e.g., ozone, microbes, volatile organic compounds (VOCs) (Malayeri et al. 2019), and aerosol particles carrying the active virus. For example, the specific types of ions were not measured in the experiment of this work, thus the future work should consider the ion species generated by the ionizers; an ultrasonic nebulizer should be considered to uniformly generate the particles from polystyrene latex sphere (PSL) in the experiment (Shiue et al. 2011). The impact of ionizer on removing the aerosol particles settling on the surfaces (walls and floors) should be also investigated in future work, because of the reflection effects of particles. The evaporation of exhaled droplets by infected occupants and the influence of various environmental factors, such as temperature and humidity (Zhu et al. 2021), should be further considered in the simulations. The releasing time of ionizers, life span of negative ions and dynamic property of natural ventilation affect the suspended negative ion concentration in the air. Thus, the unsteady simulation model and time-dependent particle removal efficiency should be considered. In general, the optimal layout strategy and parameters of ionizers should be refined under different scenarios of a real-life indoor environment (considering different room types, sizes, and indoor ventilation conditions). Although the combined effect of ionizer and natural ventilation system was evaluated by Eqs. (4) and (5) in this work, the contribution (ratio or coefficient) of ionizer and natural ventilation to the synergistic effect (such as reduction of pollutant concentration, removal efficiency, and infection risk) should be further considered in the future work. The comparisons of removal efficiencies among using ionizers, UV lamps, and air filters can be investigated to improve the comprehensive removal performance of pollutants, particularly during the regular prevention and control of the epidemic (Feng et al. 2021b).

### 5 Conclusions

This work mainly used a simulation methodology to investigate the diffusion of negative ions and the removal performance of particles in the naturally ventilated classroom under different scenarios of negative ion generators (number and location) and infected students. The optimal layout strategy of ionizers was obtained by comprehensively evaluating the removal efficiency and infection risk. The main conclusions are as follows.

(1) The simulation agreed well with the experiment, with the largest difference of 8.5% and 14.3% for velocity and particle concentration, respectively, and an average difference of 2% for negative ions. The average removal efficiency of an ionizer was approximately 70% in the indoor chamber.

(2) The coverage percentage of negative ions in the desk area in the classroom reached higher than 60% when the number of ionizers was at least 4; the maximum negative ion concentration could be 5 million #/cm$^3$. Compared with the particle concentration when no ionizer was installed, the particle concentration at the breathing plane of 1.1 m when 5 ionizers were installed could be largely reduced by 51%, when combining the natural ventilation.

(3) Through combining the ionizers and natural ventilation, the average removal efficiency was up to 85% and the average infection risk was largely reduced by 23%, when the ionizer number was 5 and the location was upstream of the classroom. This setup is the optimal layout strategy for ionizers, particularly when the infected student's location changes.

**Electronic Supplementary Material (ESM):** the Appendix is available in the online version of this article at https://doi.org/10.1007/s12273-022-0959-z.

### Acknowledgements

The authors would like to acknowledge the supports from the National Natural Science Funds for Distinguished

Young Scholar (No. 52225005), and the National Natural Science Foundation of China (No. 52178069), and the Fundamental Research Funds for the Central Universities (No. 4301002171), and Concordia University-Canada, through the Concordia Research Chair-Energy & Environment, and the support received through the Engineering and Physical Sciences Research Council (EPSRC) – funded CO-TRACE (COvid-19 Transmission Risk Assessment Case studies-Education Establishments, EP/W001411/1) and the COVAIR (Is SARS-CoV-2 airborne and does it interact with particle pollutants?, EP/V052462/1) projects, and "Knowledge Transfer and Practical application of research on Indoor Air Quality (KTP-IAQ)" project that is funded by the University of Surrey's Research England funding under the Global Challenge Research Fund (GCRF).

### Declaration of competing interest

The authors have no competing interests to declare that are relevant to the content of this article.

### Ethical approval

This study does not contain any studies with human or animal subjects performed by any of the authors.

### Author contribution statement

All authors contributed to the study conception and design. Material preparation, data collection and analysis were performed by Fariborz Haghighat, Zhuangbo Feng and Prashant Kumar. The first draft of the manuscript was written by Chen Ren and Shi-Jie Cao and all authors commented on previous versions of the manuscript. All authors read and approved the final manuscript.

**Open Access**: This article is licensed under a Creative Commons Attribution 4.0 International License, which permits use, sharing, adaptation, distribution and reproduction in any medium or format, as long as you give appropriate credit to the original author(s) and the source, provide a link to the Creative Commons licence, and indicate if changes were made.

The images or other third party material in this article are included in the article's Creative Commons licence, unless indicated otherwise in a credit line to the material. If material is not included in the article's Creative Commons licence and your intended use is not permitted by statutory regulation or exceeds the permitted use, you will need to obtain permission directly from the copyright holder.

To view a copy of this licence, visit http://creativecommons.org/licenses/by/4.0/

## Electronic Supplementary Material

Appendix to: Impact of ionizers on prevention of airborne infection in classroom
