# Supplementary material for: Impact of ionizers on prevention of airborne infection in classroom
Source: Build Simul. 2022 Dec 2;16(5):749–64. doi: 10.1007/s12273-022-0959-z (PMC9716175; doi:10.1007/s12273-022-0959-z)
Supplement: Supplementary file 1 — Appendix to: Impact of ionizers on prevention of airborne infection in classroom [file 12273_2022_959_MOESM1_ESM.pdf]

Electronic Supplementary Material

Appendix to: Impact of ionizers on prevention of airborne infection in classroom

Chen Ren[1], Fariborz Haghighat[2,1], Zhuangbo Feng[1], Prashant Kumar[3,4,1], Shi-Jie Cao[1,3] (✉)

1. School of Architecture, Southeast University, 2 Sipailou, Nanjing 210096, China
2. Energy and Environment Group, Department of Building, Civil and Environmental Engineering, Concordia University, Montreal, H3G 1M8, Canada
3. Global Centre for Clean Air Research (GCARE), School of Sustainability, Civil & Environmental Engineering, Faculty of Engineering & Physical Sciences, University of Surrey, Guildford GU2 7XH, Surrey, UK
4. Institute for Sustainability, University of Surrey, Guildford GU2 7XH, Surrey, UK

Supporting information to https://doi.org/10.1007/s12273-022-0959-z

Appendix A Diagrams of ionizer and on-site negative ion measurement

Figure A1 shows the photo of experimental setup in the indoor chamber, including air conditioner, thermal anemometer, air quality meter, air ion counter, negative ion generator (ionizer) and particle release device. Figure A2 shows the diagrams of the on-site measurement of ion generation rate using an air ion counter (Alphalab Inc AICZX21), and the ionizer device used in this work. The unit of this air ion counter is 1 million ions per cm^3. The ion generation rate of an ionizer was measured with the measurement distance of 0.1 m between the air ion counter and ionizer. The measurement period was set as 30 min, and the average value of negative ion generation rate was measured as 50 million $\#/cm^3$, and positive ion generation was neglected. The ion counter was also used to record the negative ion concentrations at the monitoring locations at different heights in the indoor chamber.

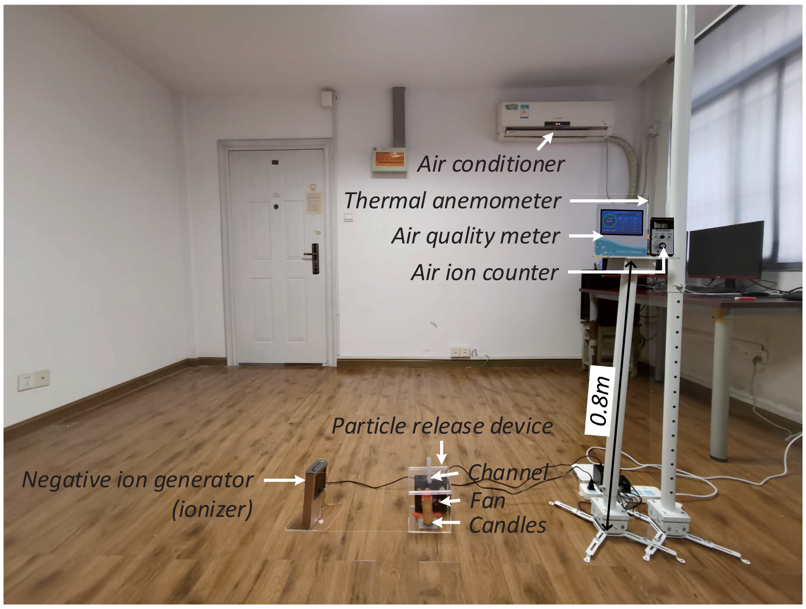

Fig. A1 Photo of experimental setup in the indoor chamber

E-mail: shijie_cao@seu.edu.cn

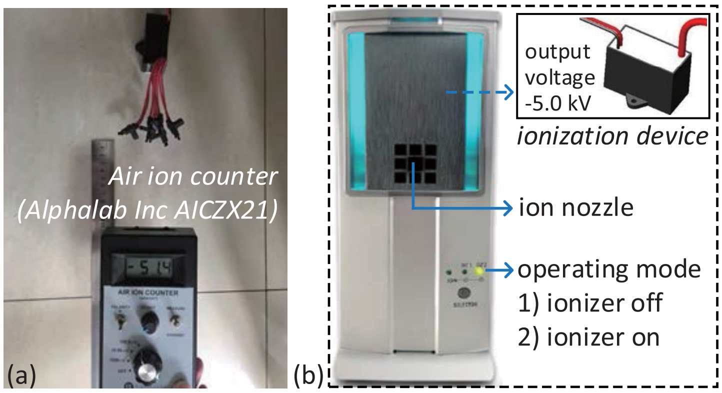

Fig. A2 Diagrams of (a) on-site measurement of ion generation rate and (b) ionizer device

Appendix B Influence of additional locations of ionizer on particle distribution, removal efficiency, and infection risk

The layout strategy of ionizers located upstream of the classroom (see Figure 5) was investigated, by analyzing the negative ion and particle distributions, removal efficiency, and infection risk. The removal performance of ionizers could be deteriorated because of the limited diffusion capacity of negative ions when the distance from the ionizers increased. The additional layout strategies of ionizers (in the middle area and downstream of the classroom) were considered to analyze the influence of ionizer layouts further. Figure B1 presents the additional locations of ionizers A1–A5 and A6–A10 with the infected student at S1, S2, and S3. The overview of the simulation cases under different scenarios (G and H) of infected students and ionizers in the middle area and downstream of the classroom is listed in Table B1.

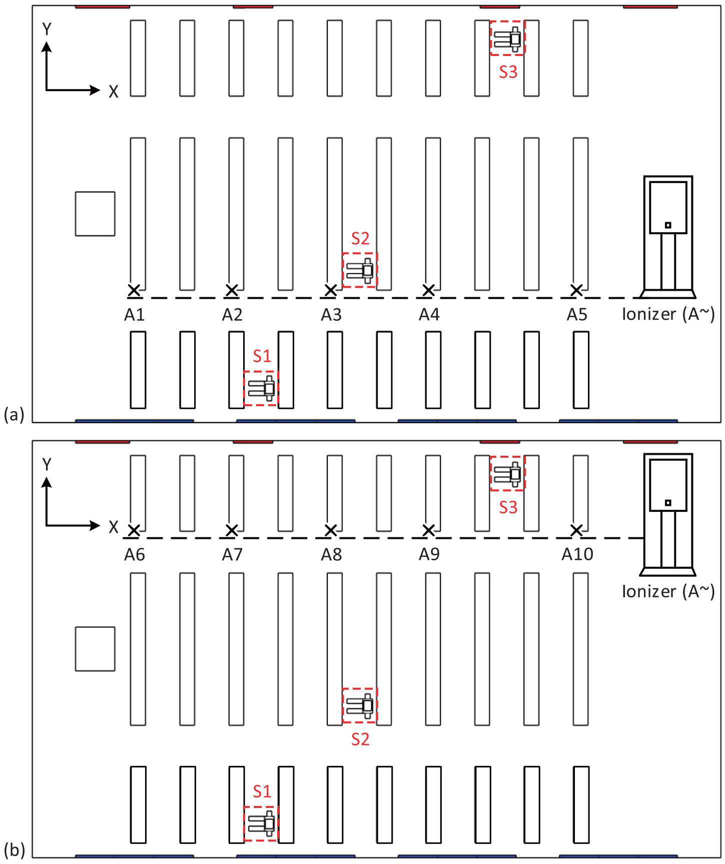

Fig. B1 Additional locations of ionizers (black **X** symbol): (a) A1–A5 in the middle area and (b) A6–A10 downstream with infected students at S1, S2, and S3 in the naturally ventilated classroom

Table B1 Overview of the simulation cases under different scenarios of infected students and ionizers in the middle area and downstream of the naturally ventilated classroom

Scenario	Case No.	Location of ionizers in the middle area and downstream of the classroom	Number of ionizers	Location of infected students
G	23–25	A1 + A2 + A3 + A4 + A5	5	S1, S2, S3
H	26–28	A6 + A7 + A8 + A9 + A10	5	Same as scenario G

Figure B2 shows the particle distribution at the plane of Z = 1.1 m when the infected student was at S1, S2, and S3, under the additional locations of the ionizers of A1–A5 and A6–A10. When 5 ionizers were installed in the middle area of the classroom and the infected student was at S1, S2, and S3, the coverage percentage of particles could reach 17.5%, 12.5%, and 25% in the desk area, respectively. The coverage percentages of particles were 27.5%, 25%, and 15% in the desk area when the ionizers were installed downstream of the classroom and the infected student was at S1, S2, and S3, respectively. The removal performance was improved when the distance away from the ionizers decreased. The finding was consistent with Figures 9–11.

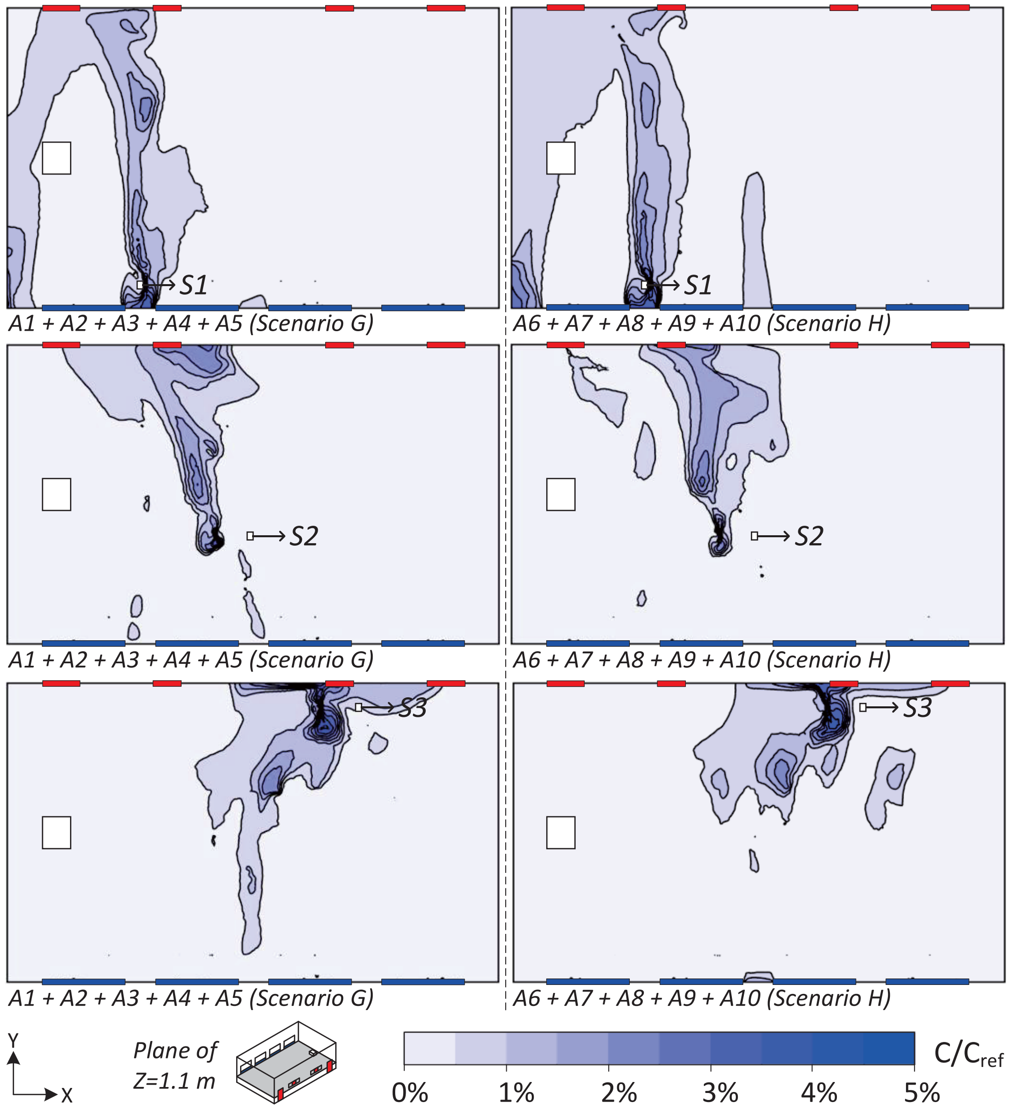

Fig. B2 Contour of relative particle concentration (C/C_{ref}) at the plane of Z = 1.1 m under infected student of S1, S2, and S3 and additional locations of ionizers (A1–A5 and A6–A10) in the naturally-ventilated classroom

Figure B3 shows the removal efficiency of ionizers (number is 5) and the infection risk when the infected student was at S1 + S2 + S3 and the additional ionizers were in the middle area and downstream of the classroom. When the ionizer location was away from the upstream, the removal efficiency decreased from 85.4% (see Figure 12) to 77.3% and 70.9%. The infection risk (with infected students at S1 + S2 + S3) when the ionizers were located downstream of the classroom largely increased by 6.1% compared with that when the ionizers were located upstream. From the perspective of removal efficiency and infection risk, placing ionizers upstream of the classroom was more favorable than placing them at the additional locations, as shown in Figure B1.

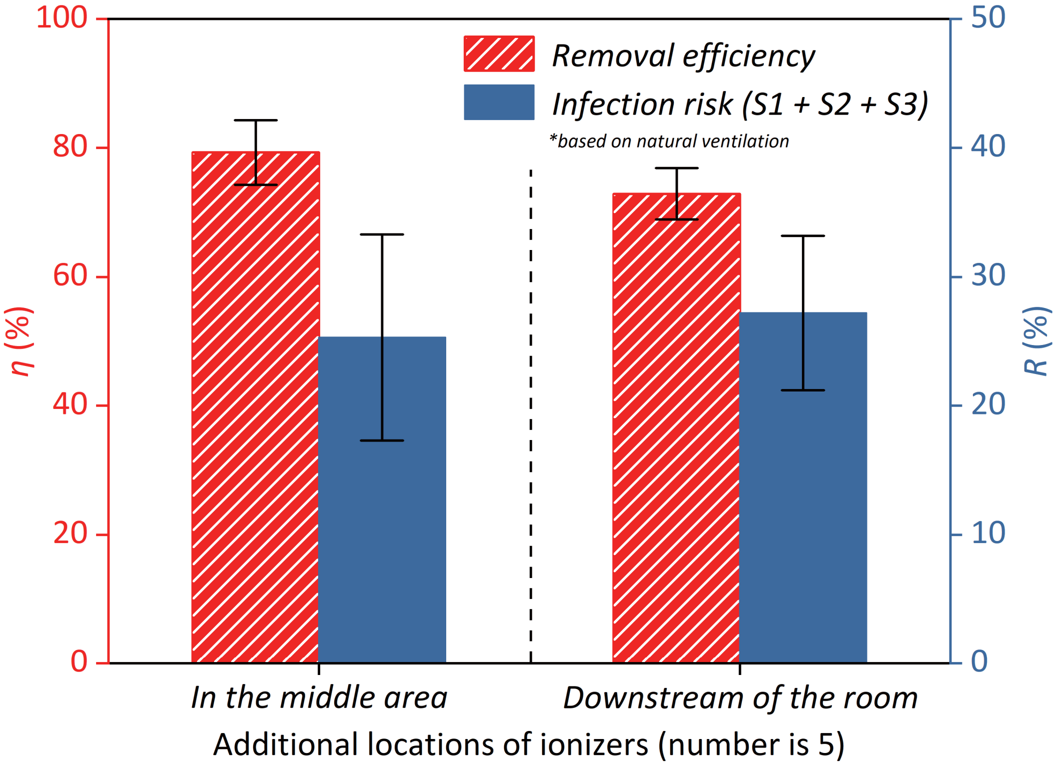

Fig. B3 Removal efficiency of ionizers (number is 5) and infection risk under infected student of S1 + S2 + S3 and additional layout locations of ionizers (in the middle area and downstream of the classroom)

Appendix C Particle distribution, removal efficiency, and infection risk without natural ventilation when independently using ionizers

To analyze the influence of independently using ionizers, the layouts of ionizers (upstream of the classroom, as shown in Figure 5) were also considered in the classroom without natural ventilation. Table C1 lists the overview of the simulation cases under different scenarios (I, J, K, L and M) of infected students and ionizers upstream of the classroom without natural ventilation. Figure C1 shows the particle distribution at the plane of Z = 1.1 m with the infected student at S1, S2, and S3 and the ionizers (with number of 1–5) upstream of the classroom without natural ventilation. Figure C2 shows the removal efficiency and infection risk of independently using ionizers under different scenarios of ionizer layouts and infected students (S1, S2, S3, and S1 + S2 + S3). Tables C2 and C3 show the significant difference analysis results based on p-values under different numbers of ionizers. Compared with the removal efficiencies when ionizers and natural ventilation were combined (in Figure 12), the removal efficiencies of ionizers used independently were reduced with a maximum percentage of 48% (with the ionizer number of 5 and infected student at S1). The infection risk of independently using ionizers was reduced when the number of ionizers was increased. However, the minimum infection risk with ionizers used independently was still larger than that with the natural ventilation used independently. Thus, combining the ionizers and natural ventilation is necessary.

Table C1 Overview of the simulation cases under different scenarios of infected students and ionizers (upstream) in the classroom without natural ventilation

Scenario	Case No.	Location of ionizers upstream of the classroom without natural ventilation	Number of ionizers	Location of infected students
I	29–31	G4	1	S1, S2, S3
J	32–34	G3 + G6	2	Same as scenario I
K	35–37	G1 + G3 + G6	3	Same as scenario I
L	38–40	G1 + G3 + G6 + G8	4	Same as scenario I
M	41–43	G1 + G2 + G4 + G6 + G8	5	Same as scenario I

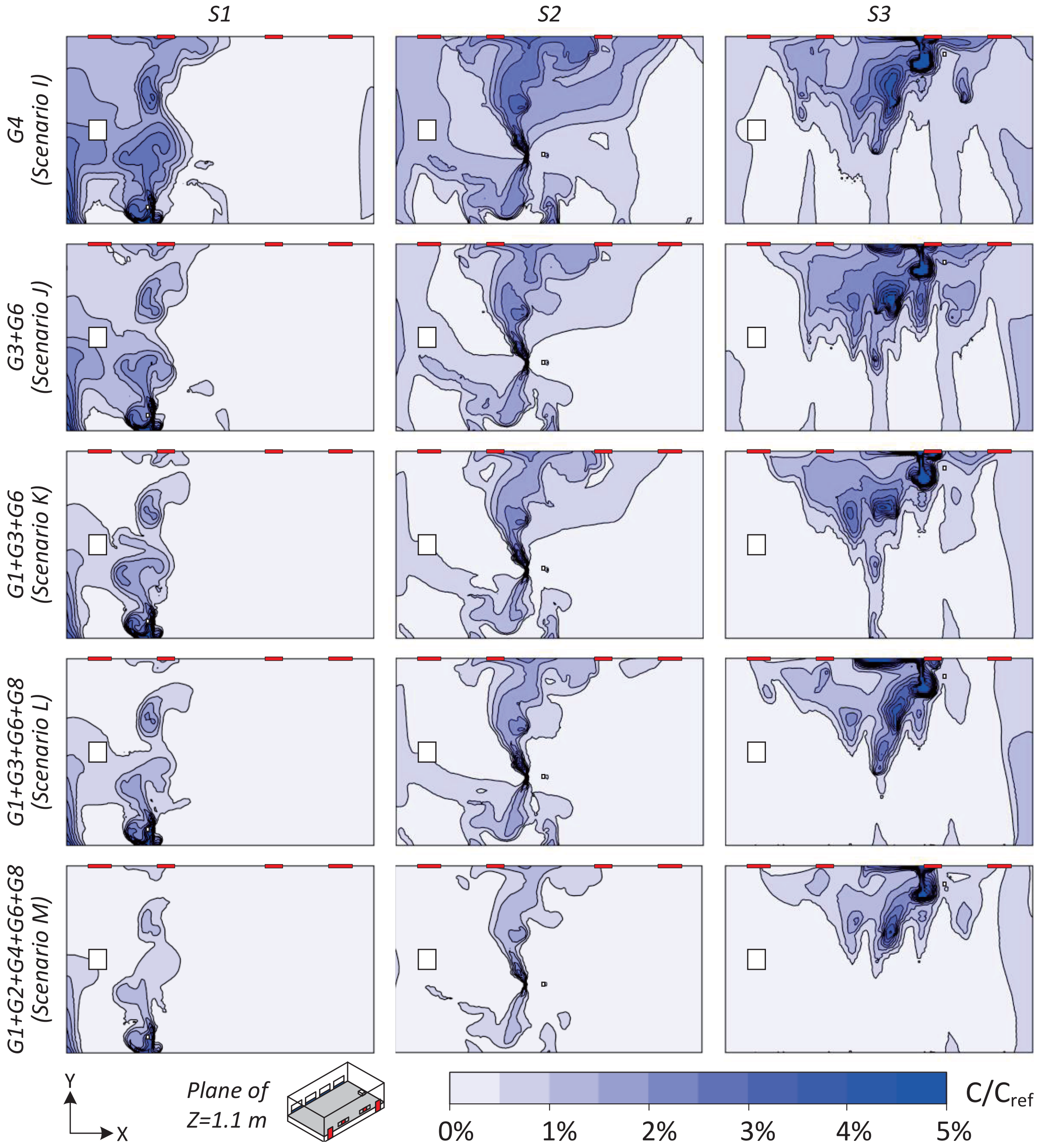

Fig. C1 Contour of relative particle concentration (C/C_{ref}) at the plane of Z = 1.1 m under infected student of S1, S2, and S3 and different scenarios of ionizers upstream of the classroom without natural ventilation

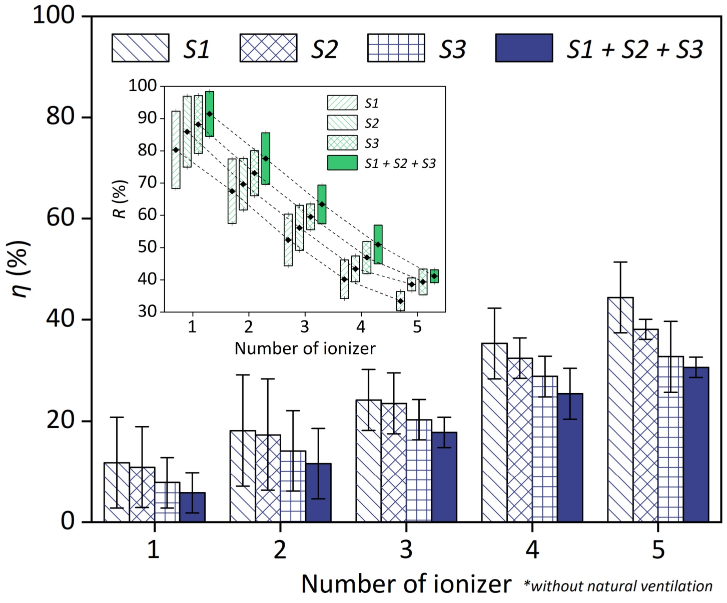

Fig. C2 Removal efficiency and infection risk of independently using ionizers under different numbers of ionizers (1–5) upstream of the classroom and infected students of S1, S2, S3, and S1 + S2 + S3

Table C2 Significant difference analysis based on *p*-value of removal efficiency under various numbers of ionizers (1–5) when independently using the ionizers

Number of ionizers	1	2	3	4	5
1	1	0.05	2.44E−05	1.79E−08	1.28E−09
2		1	2.53E−03	8.48E−05	3.36E−06
3			1	5.46E−02	7.24E−04
4				1	4.83E−02
5					1

Table C3 Significant difference analysis based on *p*-value of infection risk under various numbers of ionizers (1–5) when independently using the ionizers

Number of ionizers	1	2	3	4	5
1	1	5.74E−04	2.42E−08	1.59E−11	8.61E−14
2		1	4.58E−04	6.47E−08	1.75E−11
3			1	2.95E−05	6.31E−09
4				1	9.46E−05
5					1

Appendix D Removal efficiency with two infected students (S1 + S2, S1 + S3, and S2 + S3) and different numbers of ionizers

The removal efficiency with two infected students (S1 + S2, S1 + S3, and S2 + S3) and different numbers of ionizers (0–5) is demonstrated in Figure D1. Table D1 displays the significant difference analysis results based on *p*-values under different numbers of ionizers (0–5). The removal efficiency was obtained based on the particle concentrations in the breathing area of $Z \leq 1.1$ m. The removal efficiency increased linearly as the ionizer number increased. When the number of ionizer was 0 and 1, the average removal efficiencies were below 20% with two infected students. The average removal efficiency was increased as the number of ionizers increased to 2, 3, 4, and 5. Five ionizers could contribute to the comprehensive removal of particles, particularly when the location of the infected student changed.

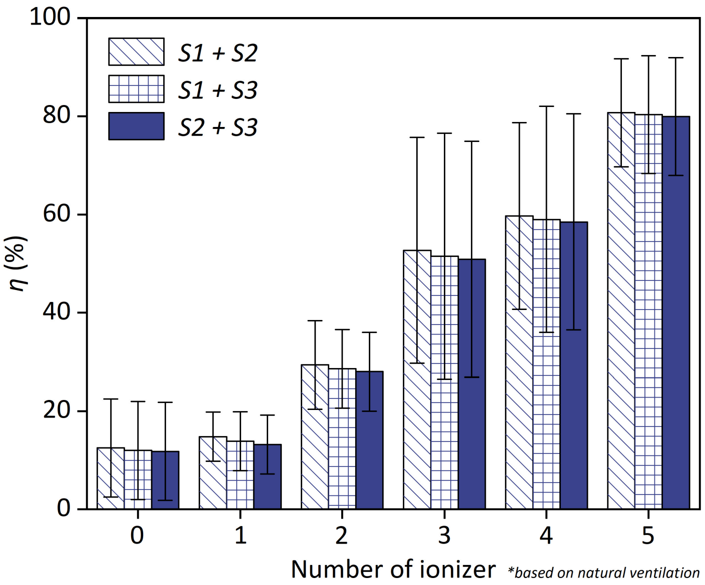

Fig. D1 Removal efficiency under different numbers of ionizers (0–5) at the upstream area and different scenarios of two infected students (S1 + S2, S1 + S3, and S2 + S3) in the naturally-ventilated classroom

Table D1 Significant difference based on *p*-value of removal efficiency under different numbers of ionizers (0–5) and different scenarios of two infected students (S1 + S2, S1 + S3, and S2 + S3)

Number of ionizers	0	1	2	3	4	5
0	1	0.58	4.52E−04	7.63E−05	3.67E−06	5.36E−11
1		1	2.13E−02	4.96E−04	8.32E−05	6.84E−09
2			1	2.37E−02	3.74E−04	5.12E−06
3				1	2.56E−02	4.25E−04
4					1	7.53E−03
5						1
